# A Novel Metastable Pentavalent Plutonium Solid Phase on the Pathway from Aqueous Plutonium(VI) to PuO_2_ Nanoparticles

**DOI:** 10.1002/anie.201911637

**Published:** 2019-11-06

**Authors:** Kristina O. Kvashnina, Anna Yu. Romanchuk, Ivan Pidchenko, Lucia Amidani, Evgeny Gerber, Alexander Trigub, Andre Rossberg, Stephan Weiss, Karin Popa, Olaf Walter, Roberto Caciuffo, Andreas C. Scheinost, Sergei M. Butorin, Stepan N. Kalmykov

**Affiliations:** ^1^ Institute of Resource Ecology Helmholtz Zentrum Dresden-Rossendorf (HZDR) PO Box 510119 01314 Dresden Germany; ^2^ The Rossendorf Beamline at ESRF The European Synchrotron, CS40220 38043 Grenoble Cedex 9 France; ^3^ Department of Chemistry Lomonosov Moscow State University 119991 Moscow Russia; ^4^ National Research Centre “Kurchatov Institute” 123182 Moscow Russia; ^5^ Directorate for Nuclear Safety and Security European Commission, Joint Research Centre Postfach 2340 76215 Karlsruhe Germany; ^6^ Department of Physics and Astronomy Molecular and Condensed Matter Physics Uppsala University P.O. Box 516 Uppsala Sweden

**Keywords:** actinide chemistry, electronic-structure calculations, pentavalent plutonium, plutonium dioxide nanoparticles, Pu M_4_ HERFD

## Abstract

Here we provide evidence that the formation of PuO_2_ nanoparticles from oxidized Pu^VI^ under alkaline conditions proceeds through the formation of an intermediate Pu^V^ solid phase, similar to NH_4_PuO_2_CO_3_, which is stable over a period of several months. For the first time, state‐of‐the‐art experiments at Pu M_4_ and at L_3_ absorption edges combined with theoretical calculations unambiguously allow to determine the oxidation state and the local structure of this intermediate phase.

## Graphical abstract

**Solid proof**: The formation of a novel pentavalent plutonium solid phase was observed on the pathway from aqueous Pu^VI^ to PuO_2_ nanoparticles by applying a high energy resolution fluorescence detection method at the Pu M_4_ and L_3_ edges and electronic structure calculations.

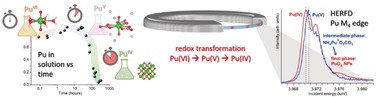

Plutonium plays a prominent role in nuclear energy production but nuclear accidents and nuclear weapons tests have led to the release of Pu and other hazardous isotopes into the environment in the past, and Pu contamination has been detected in waters and soils.^1^ Based on such cases, several countries decided to shut down the operation of the oldest nuclear facilities and put effort into improving the safety of nuclear waste storage in order to prevent further release of radioactive nuclides into the environment. To progress in this direction, it is fundamental to deepen our basic knowledge of the chemistry of actinides in environmentally relevant conditions by making compounds, characterizing them, and understand them experimentally and theoretically. Thanks to the increased experimental sensitivity, recent cross‐activities between theory and experiment, and different synthetic approaches, such a goal becomes reachable.

In spite of the low solubility of the most prevalent environmental species, Pu has been shown to be transported by groundwater from contaminated sites for several kilometers in the form of colloids, with Pu being absorbed on clays,^2^ iron oxides,^3^ or natural organic matter.^4^ In the near‐field conditions of geological repositories of spent nuclear fuel and other radioactive wastes, the formation of intrinsic PuO_2_ colloids is a key scenario.^5^ Therefore, the characterization of such intrinsic colloidal nanoparticles (NPs) in aqueous solution has recently received much attention.^6^, ^7^, ^8^, ^9^, ^10^ The most debated question is the structural nature of these NPs (crystalline vs. amorphous) as well as the presence of Pu^V^ and other oxidation states in small NPs (<3 nm).^9^, ^11^, ^12^, ^13^, ^14^, ^15^ Various studies used different synthetic approaches and different solution conditions to examine a precipitated product, either amorphous or crystalline. This has led to a controversy which has not been resolved. For example, Walther et al.^14^ observed evidence for multiple Pu oxidation states (III, IV, V) in the early stages of hydrolysis and polymerization of PuO_2_ colloids at pH 0.5–1.0, while Rothe et al.^9^ reported Pu^IV^ oxyhydroxide‐colloid formation. Conradson et al.^11^ examined solid precipitates prepared by a variety of synthetic approaches and argued for the presence of Pu^V^ in nonstoichiometric PuO_2+*x*_ solids.

One of the most fundamental properties of the chemical behavior of Pu is the variety of its oxidation states. The oxidation state is defined by the number of electrons that are removed from the valence orbitals of a neutral atom. In the pentavalent oxidation state, Pu has three electrons in the 5f shell, leaving the 6d orbitals empty. The oxidation state of Pu determines its chemical behavior and reactivity. Four oxidation states (from III to VI) may co‐exist under environmental conditions, while oxidation states VII and VIII are proposed to be stable under highly alkaline oxidative conditions.^16^ Oxidation states of aqueous, solid‐state, and interfacial Pu species have been previously determined using Pu L_3_ edge^6^, ^7^, ^17^ X‐ray absorption near edge structure (XANES) spectroscopy. The Pu edge of the L_3_ XANES spectrum of Pu^V^ always shows a characteristic energy shift towards low energies compared to Pu^IV^ and Pu^VI^ XANES spectra. The experimental energy resolution of the recorded XANES data can be improved if the spectra are recorded in the high energy resolution fluorescence detection (HERFD) mode.^8^ Nevertheless, at the Pu L_3_ edge, the electrons are excited from the 2p core level to the 6d level, which is always unoccupied independent of the Pu oxidation state. For uranium systems, we have previously shown that HERFD experiments at the U M_4_ edge^18^, ^19^, ^20^ are much more informative on the oxidation state and electronic structure than measurements at the L edges. X‐ray absorption at the M_4_ edge of actinides probes 5f states via transitions from the 3d core level. To our knowledge, HERFD data at the Pu M_4_ edge have never been reported in the literature and have never been exploited.

Figure 1 a shows the first experimental HERFD data at the Pu M_4_ edge for the Pu^IV^O_2_ and KPu^V^O_2_CO_3(s)_ (solid) systems with Pu^IV^ and Pu^V^ oxidation states, respectively. Data were collected with an X‐ray emission spectrometer^21^ set to the maximum of the Mβ emission line at 3534 eV. Synthesis procedures and the characterization of both materials are reported in the Supporting Information. The HERFD spectrum of PuO_2_ clearly shows two intense peaks, at ≈3970.2 eV and ≈3971.8 eV. According to the results of calculations carried out in the framework of the Anderson impurity model (AIM; Figure 1 b),^22^, ^23^, ^24^ the intensity and energy of these two peaks are a result of multiple factors, such as the strength of the intra‐atomic and crystal‐field interactions, and the degree of the Pu 5f/ligand 2p hybridization in the ground and final states of the spectroscopic process. In comparison with PuO_2_, the HERFD spectrum of KPuO_2_CO_3(s)_ shifts towards higher incident energies and shows a narrow profile with an asymmetric shape and a shoulder at the higher incident energy side. The results of the AIM calculations reported in Figure 1 b show a good agreement with the experimental KPuO_2_CO_3(s)_ HERFD spectrum, confirming the presence of the pentavalent Pu oxidation state in KPuO_2_CO_3(s)_.

**Figure 1:**
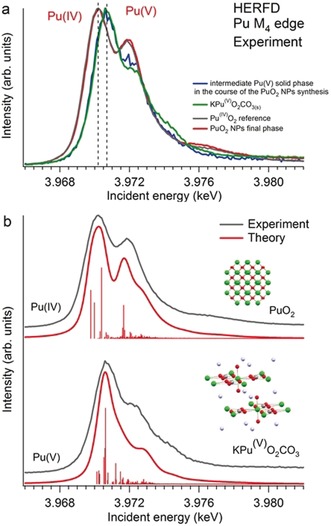
a) Experimental HERFD data at the Pu M_4_ edge from two plutonium phases obtained during the synthesis of PuO_2_ nanoparticles (NPs) from a Pu^VI^ precursor at pH 11. Blue curve: spectrum of the intermediate Pu^V^ solid phase appearing during the synthesis of the PuO_2_ NPs; red curve: spectrum of the final phase of PuO_2_ NPs. The spectra of a PuO_2_ bulk sample (grey curve) and of KPuO_2_CO_3(s)_ (green curve) are also shown as references for Pu^IV^ and Pu^V^ oxidation states, respectively. Data were collected with an X‐ray emission spectrometer set to the maximum of the Mβ emission line at 3534 eV. b) Experimental HERFD spectra of PuO_2_ and KPuO_2_CO_3(s)_ compared with the results of Anderson impurity model calculations.

Due to dipole selection rules (*J=*0;±1), the shape of the Pu M_4_ and M_5_ HERFD transitions is expected to be different. At the Pu M_5_ edge, the unoccupied 5f electronic levels with *J=*5*/*2 and 7*/*2 can be reached by an electron excited from the Pu 3d_5/2_ state, whereas only the *J=*5*/*2 state can be reached at the Pu M_4_ edge.^25^ A comparison between Pu M_4_ and Pu M_5_ spectra for several Pu systems is shown in Figure S1 (Supporting Information). The energy shifts between Pu^III^, Pu^IV^, and Pu^V^ in solid compounds are found to be in the order of 2 eV (between Pu^III^ and Pu^IV^) and 0.4 eV between Pu^IV^ and Pu^V^ (Table S1). A correct determination of the Pu oxidation state therefore requires the improved energy resolution of the absorption spectra provided by HERFD.

Figure 1 a shows experimental HERFD data recorded at different stages during the synthesis of PuO_2_ NPs from the aqueous Pu^VI^ precursor. For this purpose, a solution of Pu^VI^ was added to an excess of ammonia. The measured pH value of the solution was 11. We kinetically traced the route of the Pu^VI^‐to‐PuO_2_ transformation as a two‐step process: during the first minutes, we observed the formation of an intermediate Pu phase consisting of yellow sludge (see Figure 2). Later, during the formation of PuO_2_ NPs, the intermediate phase dissolved and a different equilibrium phase (named “final phase” in the following) was formed.^26^ The Pu M_4_ HERFD spectrum recorded at the intermediate stage of the reaction is represented by the blue curve in Figure 1 a. The spectrum clearly indicates the presence of the Pu^V^ oxidation state. This is supported by the good correspondence between energy and the relative intensities of the main features of the Pu M_4_ edge spectrum for KPuO_2_CO_3(s)_ (green curve) and the Pu intermediate phase (blue curve).

**Figure 2:**
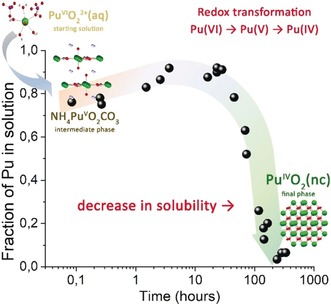
Kinetics of the precipitation of Pu starting from Pu^VI^ at pH 11 ([Pu]=6×10^−5^ m). Inset: Crystal structure of the formed phases.

Furthermore, the HERFD spectrum of the final product of the reaction, formed after 3 weeks of the precipitation reaction, shows an identical profile to the one detected for PuO_2_ single crystal, confirming that the reaction terminates with the formation of PuO_2_ NPs with cubic structure and with the Pu^IV^ oxidation state, as reported by Soderholm et al.^15^ for Pu_38_ clusters (Li_14_(H_2_O)_*n*_[Pu_38_O_56_Cl_54_(H_2_O)_8_]) isolated from the initially alkaline peroxide solution.^15^

The experimental data collected for the intermediate phase during the PuO_2_ NPs synthesis show evidence of the Pu^V^ oxidation state. The exact contribution of the different chemical states in the Pu M_4_ HERFD data reported in Figure 1 a was estimated by the ITFA program.^27^ The results indicate that the spectrum of the intermediate Pu phase contains 87 % of Pu^V^ and 13 % of Pu^IV^ (with an estimated root‐mean‐square error of less than 2 %, see Figure S2). We did neither observe a significant contribution of Pu^V^ in the final phase (after the PuO_2_ NPs were formed) nor a quantifiable amount of Pu^VI^ (Table S2). The absence of Pu^V^ in the final phase and the 100 % presence of the Pu^IV^ oxidation state after the PuO_2_ NPs formation is an important result. At the same time, our data demonstrate that Pu^VI^‐to‐Pu^IV^ reduction does not occur in a single step.^26^ The Pu^VI^ is first reduced to Pu^V^ and then to Pu^IV^.

Moreover, additional HERFD and EXAFS (extended X‐ray absorption fine structure) experiments at the Pu L_3_ edge gave us the opportunity to identify the intermediate phase forming in the course of the PuO_2_ NPs growth. Figure 3 shows the comparison of the Pu HERFD L_3_ edge data recorded for PuO_2_ and the intermediate Pu phase during the PuO_2_ NPs formation. As discussed previously, the L_3_ spectrum of Pu^V^ compounds always shows a very characteristic energy shift towards low energies and a decrease of the L_3_ white line intensity compared to Pu^IV^ and Pu^VI^ systems^6^, ^7^, ^8^, ^17^ (Figure S3). The chemical shift of the intermediate Pu phase is clearly resolved in the HERFD data reported in Figure 3 and indicates the presence of the Pu^V^ oxidation state, in agreement with the Pu M_4_ HERFD results. However, for actinide systems, HERFD at the L_3_ edge is not as sensitive as the M_4_ edge HERFD to the presence of minor contributions (<10 %) from different oxidation states.^28^, ^29^ HERFD at the L_3_ edge is, however, extremely sensitive to the local structure around the absorber, which results in specific post‐edge features.^19^, ^30^, ^31^

**Figure 3:**
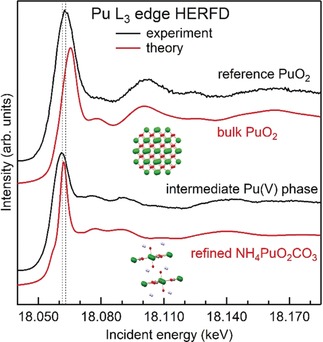
Pu L_3_ HERFD spectra of PuO_2_ and the Pu^V^ intermediate phase formed during the synthesis of PuO_2_ nanoparticles from Pu^VI^ precursors at pH 11. Experimental data (black lines) are compared with FDMNES calculations for bulk PuO_2_ and NH_4_PuO_2_CO_3_ (red lines).

Ab‐initio calculations on different structures were used to identify the intermediate Pu phase during the synthesis of the PuO_2_ NPs. We simulated the HERFD spectra of several compounds containing Pu (Figures S4 and S5) in order to determine the Pu speciation of the intermediate Pu phase structure. The best agreement is found for NH_4_PuO_2_CO_3_ in which Pu is present in the pentavalent state. The HERFD spectral shape reflects the d‐density of states (DOS) of Pu apart from the small shoulder at the absorption edge, which is barely visible in the data but well resolved in the simulation and represents the Pu 5f DOS (Figure S6). The Pu d‐DOS is involved in the bonds with O, C, and N. The Pu L_3_ EXAFS data confirmed that the intermediate Pu phase formed during the PuO_2_ NPs synthesis is compatible with NH_4_PuO_2_CO_3_. Furthermore, the EXAFS spectrum (Figure S7 and Table S3) could be fitted with a model based on the crystal structure of NH_4_PuO_2_CO_3_ that was previously published.^32^ The fitted Pu−O distance of the triple‐bond group is 1.82 Å, in good agreement with previously determined distances of 1.80–1.81 Å for Pu^V^ compounds^7^, ^17^, ^33^ (see Table S4), while the crystallographic distance of 1.93 Å is most likely biased by the very weak scattering of oxygen in comparison to Pu.^33^ We also found that the Pu−Pu coordination number in the experimental EXAFS spectra of the intermediate phase is lower than for the structural data, which can be explained either by a (partially) amorphous nature or by nano‐sized particles.

The intermediate NH_4_PuO_2_CO_3_ phase was completely dissolved within ≈10 h, after which the PuO_2_ NPs were formed as a result of longer redox reactions (see Figure 2). Finally, a part of the intermediate Pu^V^ phase was centrifuged out of suspension and dried at room temperature in order to check its stability over months. Surprisingly, the dried NH_4_PuO_2_CO_3_ phase was found to be stable over months. We recorded additional Pu L_3_ HERFD spectra after 3 months and the spectral shape remained the same (Figure S8). Therefore, the method reported here can be used to synthesize this Pu^V^ phase.

To understand the pH influence, we performed a similar experiment at pH 8. The kinetics of the Pu precipitation is very similar to the experiment at pH 11, whereas the quantity of the intermediate Pu^V^ phase is lower (Figure S9). Comparison of the experimental conditions with the available thermodynamic data shows that Eh/pH values during our synthesis correspond to the area of stability of the Pu^IV^ phase close to the phase boundary (Figure S10). It makes the formation of the intermediate Pu^V^ phase possible, but at the same time, the high thermodynamic stability of PuO_2_ and its extremely low solubility lead to a further transformation of the Pu^V^ phase into PuO_2_.

We show here for the first time that while Pu^V^ solid‐state complexes are always viewed as exotic compounds, a thermodynamically metastable Pu^V^ solid phase is formed during the reductive precipitation of PuO_2_ NPs from a Pu^VI^ precursor at pH 11. The intermediate Pu^V^ phase is characterized for the first time using HERFD at the Pu M_4_ edge and model calculations in the framework of AIM. The Pu M_4_ HERFD method allows for the unambiguous identification of the Pu oxidation state, it demonstrates the Pu^V^ existence, and provides quantitative estimates for varying Pu oxidation states. The local structure of the intermediate Pu^V^ phase, similar to NH_4_PuO_2_CO_3_, is identified by a combination of the Pu L_3_ HERFD experiment and ab‐initio calculations, and is found to be stable over a period of several months. The redox reactions behind aqueous Pu^VI^−PuO_2_ NPs and the formation of Pu^V^ cause the substantial increase of the solubility. This finding provides a significant step towards a better understanding of Pu chemistry and emphasizes the value of the HERFD technique for studies of PuO_2_ NPs formation under different conditions.

## Conflict of interest

The authors declare no conflict of interest.

## Supporting information

As a service to our authors and readers, this journal provides supporting information supplied by the authors. Such materials are peer reviewed and may be re‐organized for online delivery, but are not copy‐edited or typeset. Technical support issues arising from supporting information (other than missing files) should be addressed to the authors.

Supplementary
