# Supplementary material for: A Novel Metastable Pentavalent Plutonium Solid Phase on the Pathway from Aqueous Plutonium(VI) to PuO2 Nanoparticles
Source: Angew Chem Int Ed Engl. 2019 Nov 6;58(49):17558–62. doi: 10.1002/anie.201911637 (PMC6900038; doi:10.1002/anie.201911637)
Supplement: Supplementary file 1 — Supplementary [file ANIE-58-17558-s001.pdf]

Angewandte Chemie
Eine Zeitschrift der Gesellschaft Deutscher Chemiker

Supporting Information

A Novel Metastable Pentavalent Plutonium Solid Phase on the Pathway from Aqueous Plutonium(VI) to PuO_2 Nanoparticles

Kristina O. Kvashnina, Anna Yu. Romanchuk, Ivan Pidchenko, Lucia Amidani, Evgeny Gerber, Alexander Trigub, Andre Rossberg, Stephan Weiss, Karin Popa, Olaf Walter, Roberto Caciuffo, Andreas C. Scheinost, Sergei M. Butorin, and Stepan N. Kalmykov*

anie_201911637_sm_miscellaneous_information.pdf

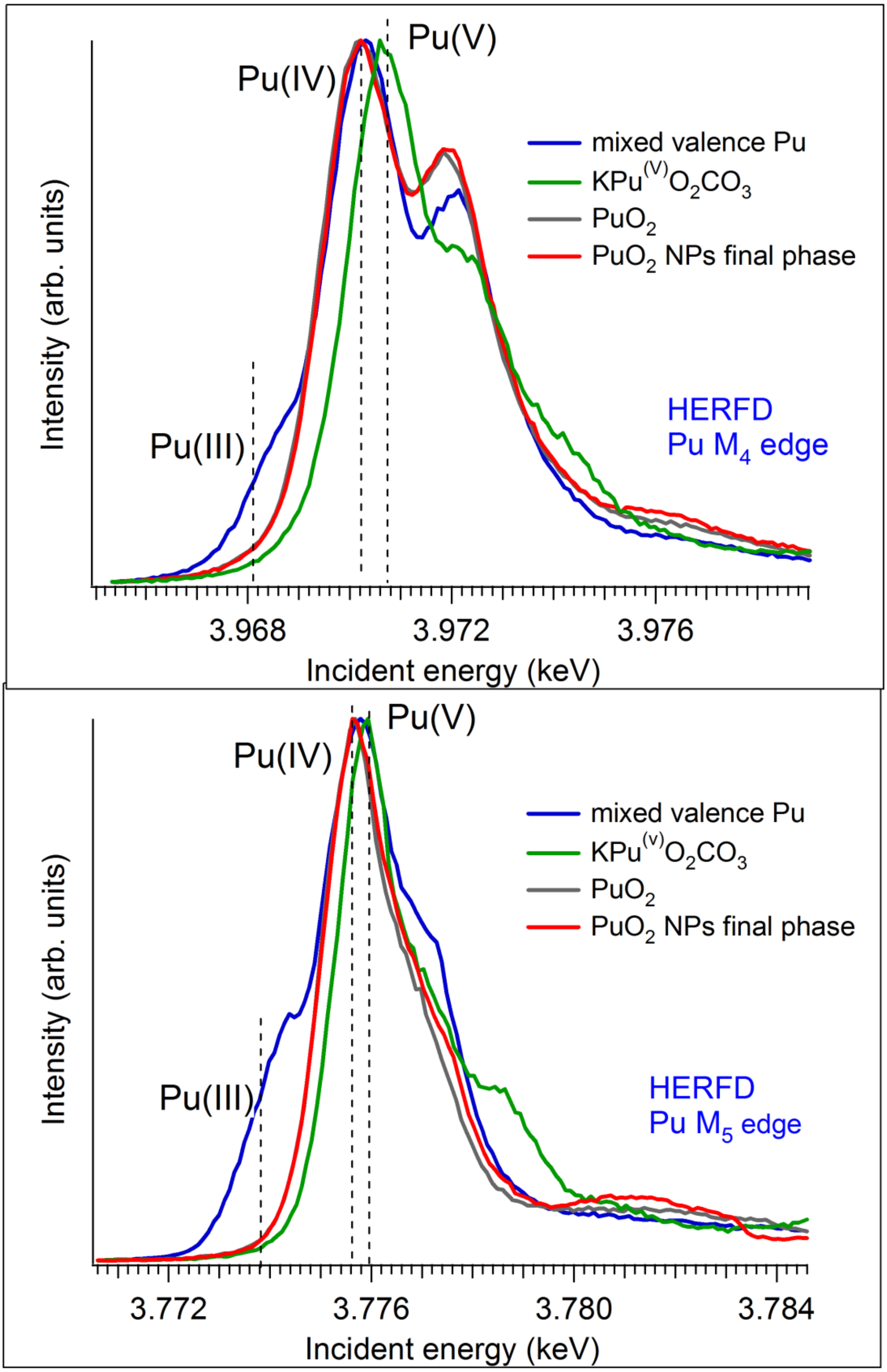

Figure S1. Pu HERFD data recorded for a number of Pu reference and PuO_2 NPs at the Pu M_4 and Pu M_5 edges. Plutonium mixed system were produced, upon decomposition of $PuO_2(NO_3)_2{*}H_2O$ system to Pu(III) and Pu(IV) oxides. The difference between PuO_2 powder sample and PuO_2 single crystal is observed, but the reason of it is beyond the scope of the present paper and wouldn't be discussed further.

Table S1: Energy position of the Pu HERFD maximum for Pu(III), Pu(IV), and Pu(V) compounds recorded at the Pu M_4 and Pu M_5 edges

	Pu(III) (eV)	Pu(IV) (eV)	Pu(V) (eV)	Recorded energy shift Pu(III) – Pu(IV) and Pu(IV)-Pu(V)
Pu M_4 edge	3968.2	3970.2	3970.7	2.0 eV and 0.4 eV
Pu M_5 edge	3773.7	3775.6	3775.9	2.0 eV and 0.3 eV

ITFA analysis:

The ITFA approach[1,2] was previously applied to studies of uranium compounds by HERFD at the U M_4 edge[3]. Here, the fractions of Pu(IV) and Pu(V) oxidation states was derived for HERFD data, recorded for two samples: the intermediate Pu phase during PuO_2 NPs formation and the final phase of PuO_2 NPs, along with two carefully characterized references, PuO_2 and $KPuO_2CO_3$. The interpolated and reproduced experimental HERFD data is reported in Fig. S2a. In the first step of the spectral analysis, a principle component analysis (PCA) was performed in order to determine the number of components contributing to the spectrum. It can clearly be seen from Fig. 2B that only the first two eigenvectors show a signal, while the following (3 and 4) consist of counting noise (ordinate) and noise in the monochromaticity; hence only two components are necessary to describe the variation in the spectral mixtures. Since we included the spectra of the two references, it is evident that the two samples consist of only these two references, which were fixed as endmembers to 100%, while their percentages in the two samples was fitted. Table S2 shows that the "intermediate Pu phase" sample contains 87% of the Pu(V) reference and 13% of the Pu(IV) reference. The final phase – PuO_2 NPs sample contains 100% of the Pu(IV) component. The iterative target test (ITT) procedure was then used to extract the noise-filtered spectrum of the two components (Fig. 2C) corresponding to the Pu(IV) and Pu(V) compounds. The ITFA analysis shows a relative concentration error of the order of 2% , according to the root mean square error (RMS).

Compound	Pu(IV)	Pu(V)
PuO_2 reference	100 %	0 %
$KPuO_2CO_{3\,(s)}$ reference	0%	100%
Intermediate Pu Phase	13%	87%
PuO_2 NPs final phase	100%	0%

Table S2: Fractions of Pu(IV) and Pu(V) contributions in the four samples: PuO_2 reference, $KPuO_2CO_3$, Intermediate Pu phase in course of PuO_2 NPs formation and final phase of PuO_2 NPs, calculated with the iterative transformation factor analysis (ITFA) method from the Pu M_4 edge HERFD spectra. The estimated root mean square error is less than 2%.

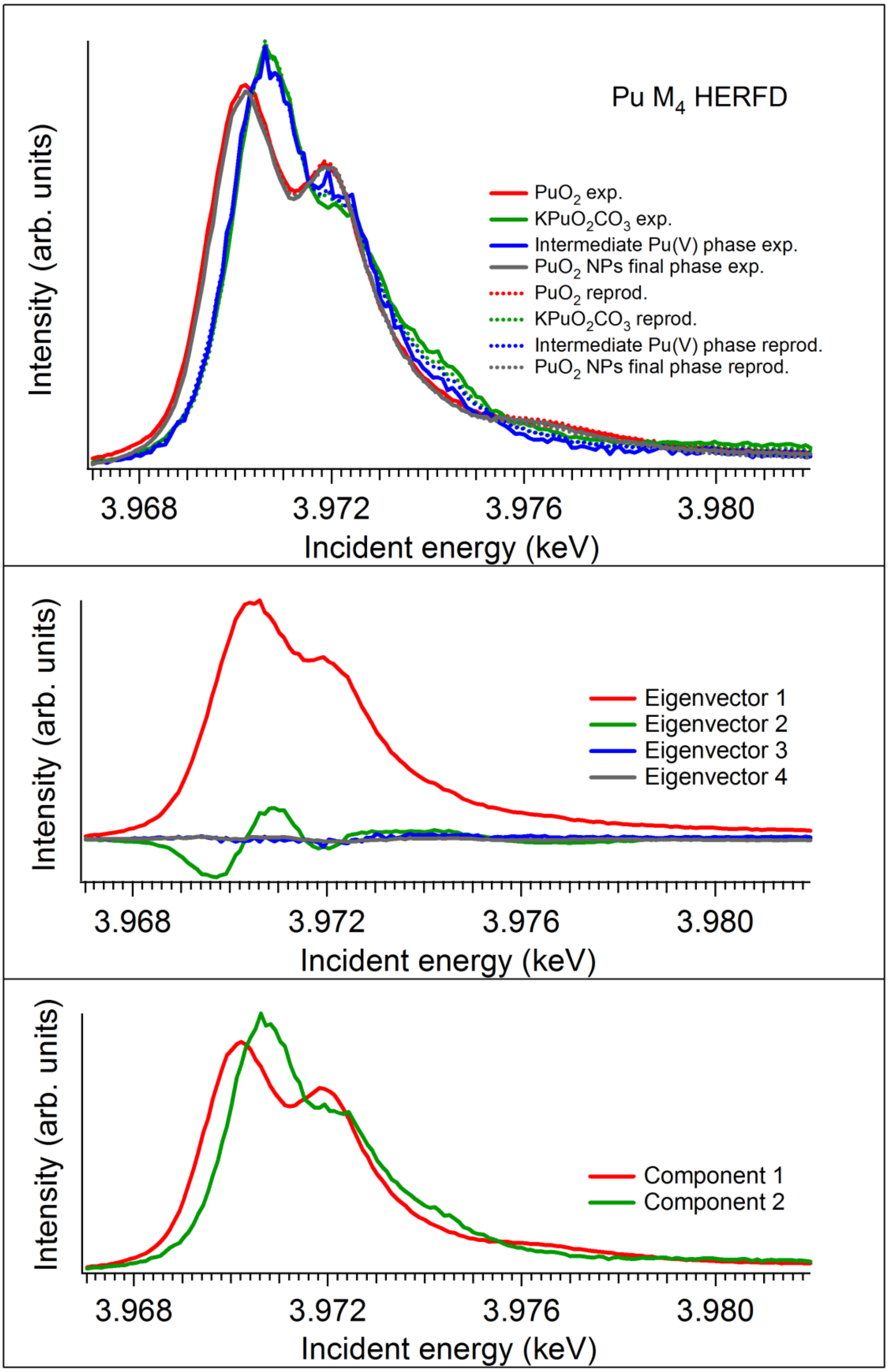

Figure S2. a) Reproduced and experimental HERFD spectra recorded at the Pu M_4 edge of PuO_2 reference, $KPuO_2CO_3$, intermediate Pu (V) phase in course of PuO_2 NPs formation and final phase of PuO_2 NPs samples computed with ITFA. b) Computed eigenvectors contributions for four samples; c) spectra of ITT-extracted components.

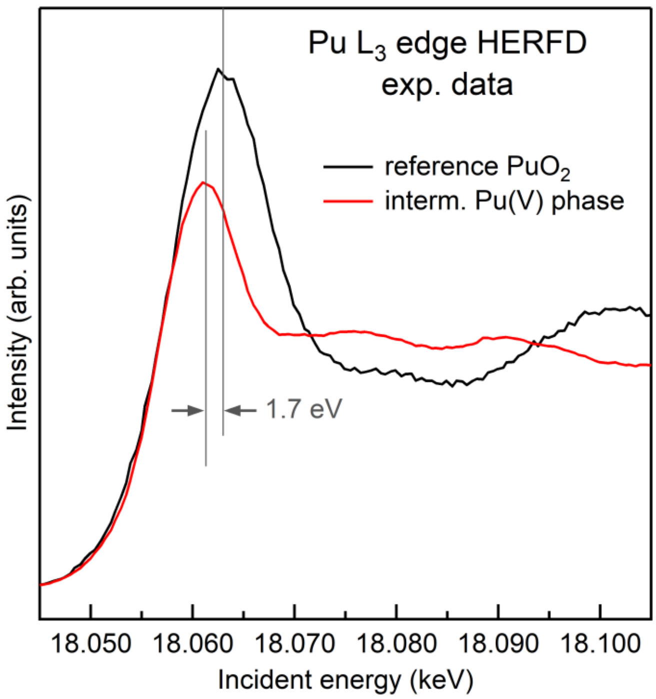

Figure S3. Experimental Pu L_3 HERFD spectra of single crystalline PuO_2 and of the Pu(V) intermediate phase formed during the synthesis of PuO_2 nanoparticles from Pu(VI) precursors at pH 11. Vertical lines indicate the energy position of the main peaks corresponding to plutonium in oxidation states V and IV (the energy difference of 1.7eV were detected in HERFD mode).

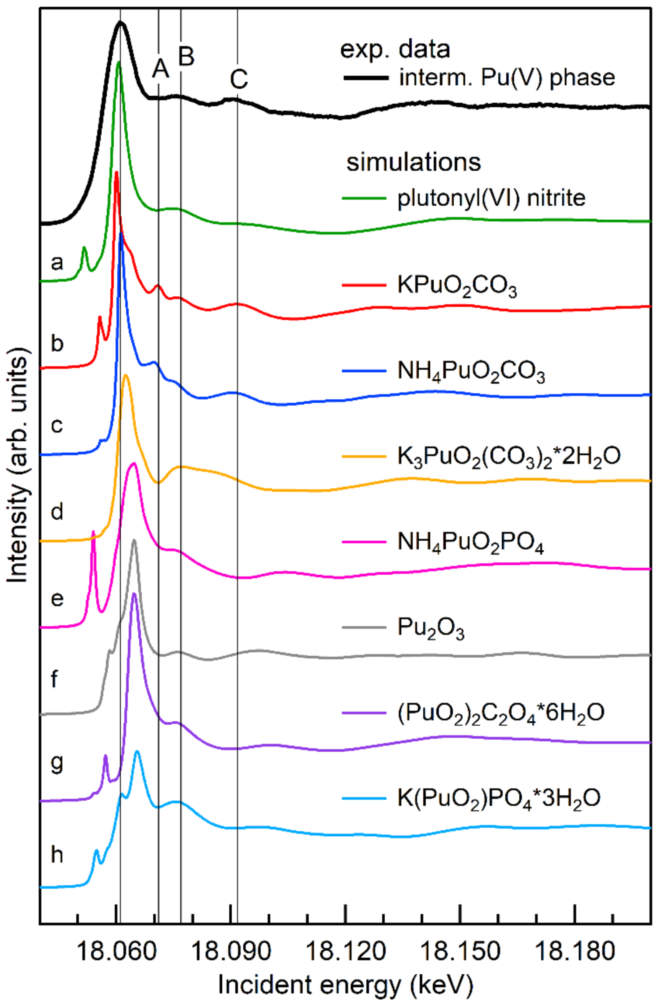

Figure S4. Comparison between the experimental Pu L3 HERFD on intermediate Pu(V) phase and FDMNES simulations of several Pu-containing compounds. The simulations adopt the Green's functions approach which is faster and allows the screening of several possible structural models. Vertical lines mark the positions of feature A, B and C of the experimental spectrum. All simulations were shifted to match the position of feature B. Structures b and c show the best agreement with the data.

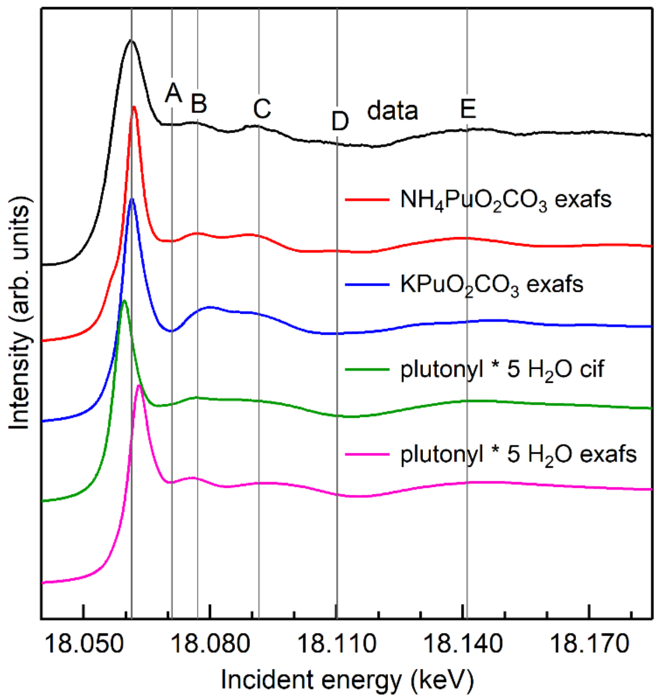

Figure S5. Pu L_3 HERFD data on the intermediate Pu(V) phase are compared to new FDMNES simulations on the most significant Pu-containing compounds. The simulations on compounds b and c of Figure S3 were improved: the shortest Pu – O bonds were changed to match the EXAFS results and the FDM method, which gives better results in the region close to the edge, was used. We also simulated with the FDM method two spectra of the plutonyl aqueous molecule: one with the shortest Pu – O bonds taken form the reported structure and one with the Pu – O bonds adapted from EXAFS results. Vertical lines mark the positions of feature A, B, and C of the experimental spectrum. Simulations were shifted in energy to match the position of feature B. The best agreement is found for $NH_4PuO_2CO_3$.

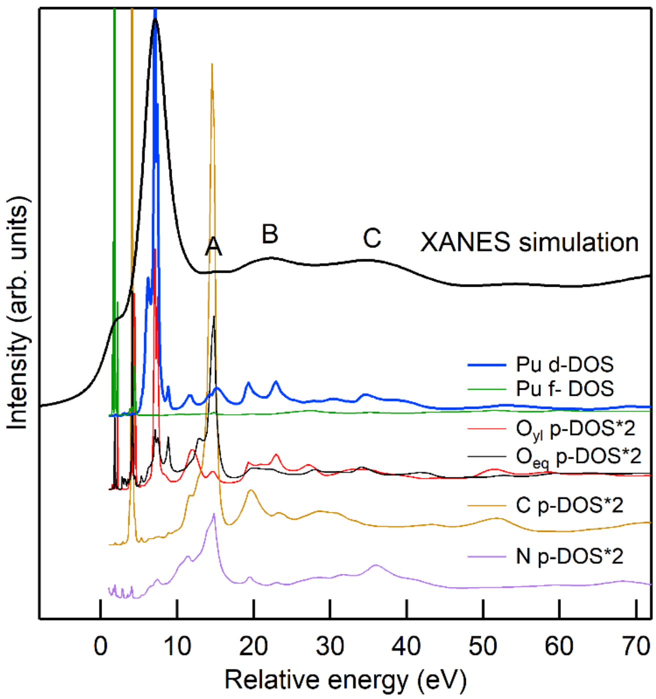

Figure S6. The simulation of $NH_4PuO_2CO_3$ shown in Figure S4 is here compared to the DOSs of Pu, O, C and N. The subscripts yl and eq are used to distinguish between the apical and the equatorial O atoms, which have very different distances from Pu.

EXAFS analysis

EXAFS data ($\chi_{exp}(k)$) were analyzed using the IFEFFIT [4]data analysis package. EXAFS data reduction used standard procedures for the pre-edge subtraction and spline background removal. The Fourier transformation (FT) of the k^3-weighted EXAFS functions $\chi_{exp}(k)$ were calculated over the ranges of photoelectron wave numbers k = 3.0–15.0 Å^{-1}. Fitting of the EXAFS spectra were performed in R-space (R-range = 1.5-5.4 Å) by varying the structural parameters, including interatomic distances (*R*), coordination numbers (*N*) and Debye–Waller factors (σ^2), were found by the non-linear fit of theoretical spectra to experimental ones.
The theoretical data were simulated using the photoelectron mean free path, amplitude and phase shift of photoelectron scattering paths calculated *ab initio* using program FEFF6 [5]. S_0^2 parameter value was set at 0.9. Intensities of scattering paths were calculated taking into account the crystal structure of $NH_4PuO_2CO_3$. Finally, while we could fit a Pu-C single scattering path at 2.93 Å suggesting carboxyl-groups coordinating to the equatorial oxygen coordination plane of Pu in bidentate fashion, we could not fit the corresponding multiple scattering path as apparent e.g. in aqueous U(V) and Np(V)[6,7] as well as in tetravalent actinide carbonate solids[8,9] suggesting a substantial distortion in this pentavalent solid.

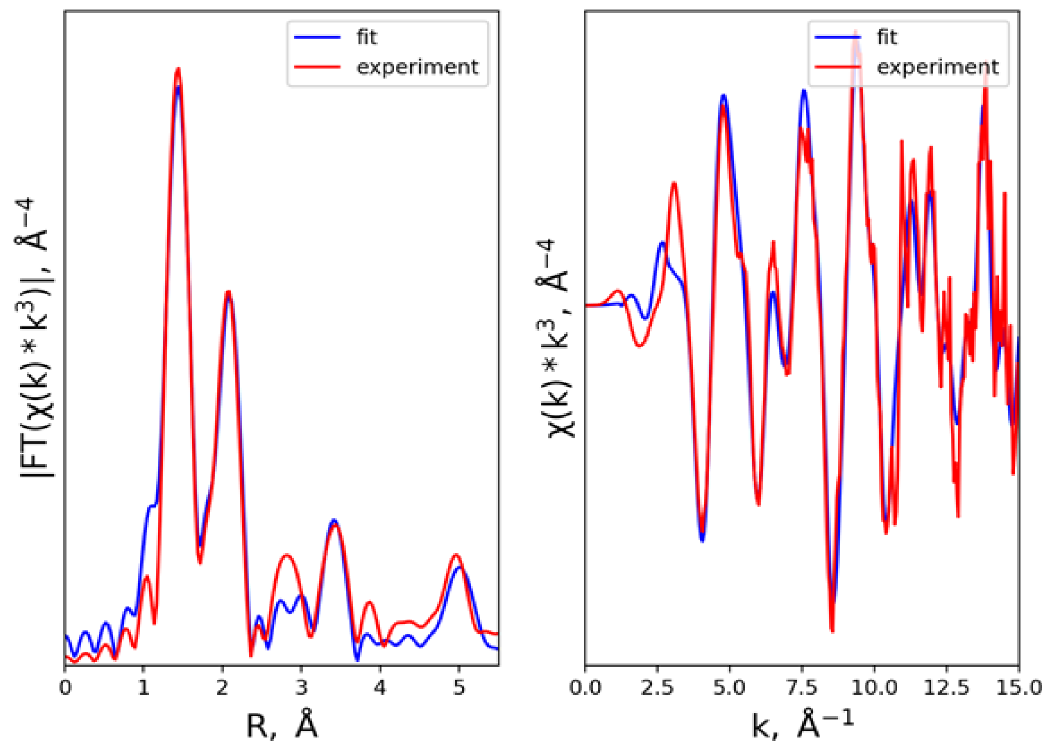

Figure S7. Pu L_3-EXAFS R-space fitting results a) FT magnitude of EXAFS data and its fitting ; b) k^3-weighted χ(k) experimental functions and corresponding fitting models

Table S3. Structural parameters obtained from the fitting of EXAFS spectra.

Sample	N	Type	R / Å	σ^2 / Å^2	Crystal structure distances and CN of $NH_4PuO_2CO_3$ from[10]	
"intermediate phase"	2*	O	1.82	0.002	1.93	2
	1.6	O	2.19	0.006		
	5.2	O	2.51	0.006	2.55	6
	0.8	C	3.01	0.002	2.93	3
	5.7	N	3.88	0.002	3.83	6
	1.3	Pu	5.15	0.002	5.09	6
	3.1	O	4.03	0.002	4.18	6

*- Fixed values

Table S4. The Pu-O distances for Pu(V) compounds reported previously in literature

Compounds	R, Å	CN	Literature
Pu(V) aqua	1.80	2	Dalodiere et al, 2018 [11]
Pu(V) aqua	1.81	2	Giandomenico et al., 2009 [12]
Pu(V) aqua	1.81	2	Conradson et al., 2004[13]

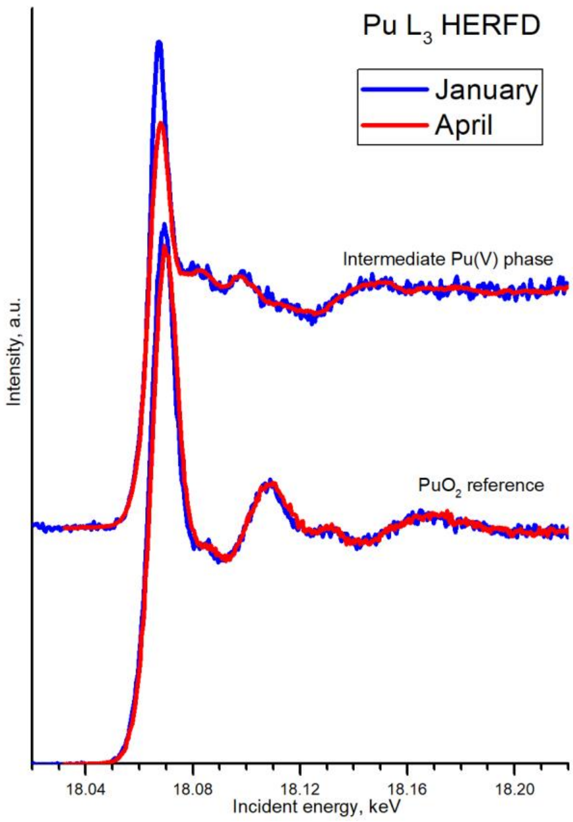

Figure S8. Pu L_3 HERFD data recorded in January 2018 and April 2018 (3 months later) on the same systems. The only difference is that HERFD data in January have been recorded with one crystal analyzer and in April with five crystal analyzers. All other conditions were identical.

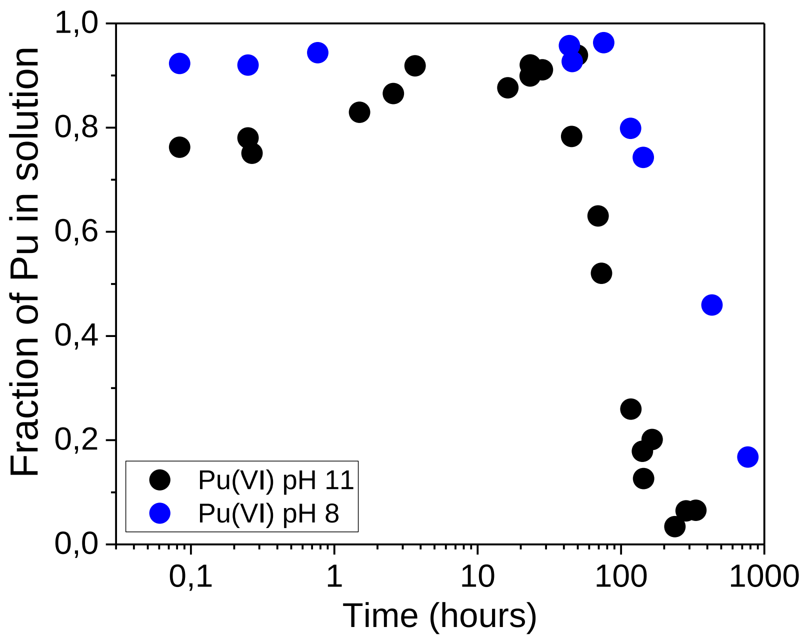

Figure S9. Kinetics of precipitation of Pu starting from Pu(VI) at different pH values ([Pu] = $6 \cdot 10^{-5}$ M)

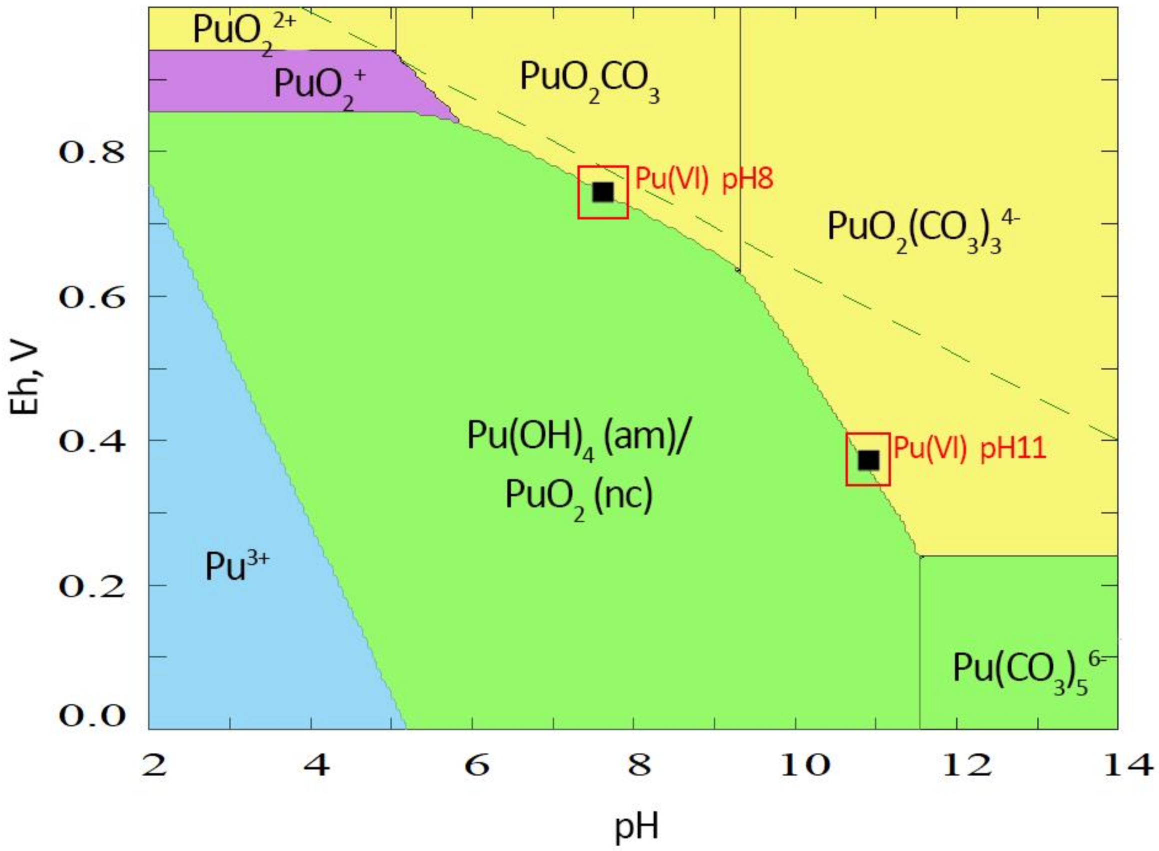

Figure S10. Pourbaix diagrams for Pu in equilibrium with p(CO2)=$10^{-3.5}$, calculated in MEDUSA software[14] ([Pu] = $6 \cdot 10^{-5}$ M, I=0.1M). The thermodynamic data from NEA database[15] was used. Unfortunately thermodynamic data for $NH_4PuO_2CO_3$ is absent in the literature.

Synthesis:

PuO_2

For M_4 edge measurements:

Pu(IV) oxalate have been produced by direct precipitation of a Pu(IV)-solution with oxalic acid and further decomposed to PuO_2 by thermal treatment (5h/ 600 C under air). Before encapsulation, the sample was thermally treated once more (2 h/ 1000 C under air).

For L_3 edge measurements:

PuO_2 reference was purchased from Oakridge National Lab (Batch I.D. No. Pu-242-327A1)

$KPuO_2CO_3$ (solid)

$KPu(V)O_2CO_{3(s)}$ was prepared as reported by Simakin et al.[16] A calculated volume of a stock Pu(IV) solution in 3.5M $HClO_4$ was evaporated nearly to dryness, half a ml of 1M $HClO_4$ was added after solution was cooled down. During this step oxidation of Pu(IV) to Pu(VI) takes place followed by a change of originally pink Pu(IV) solution to orange Pu(VI). On the next step the Pu(VI) carbonate solution was prepared by adding calculated amount of Pu(VI) into desired volume of 0.1M K_2CO_3 solution. 0.1-0.2M K_2CO_3 is reported for the preparation of $KPu(V)O_2CO_{3(s)}$ by electrochemical reduction of Pu(VI) in carbonate solutions.[1] Resulting green solution of Pu(VI) carbonate was filtered on a 0.2 µm Millipore filter and used further for the electrochemical procedure using standard three electrode scheme. The working (Pt spiral), counter (Pt spiral) and reference (Ag/AgCl) electrodes were purchased from ALS Co. Ltd. During few hour of a mild reduction, 0.1 V vs. Ag/AgCl, original green color of Pu(VI) carbonate solution fades down with a slow formation of a light-grey precipitate. The solution was then led to stay for 15 days. The resulting mixture was filtered, precipitate was washed several times with diluted K_2CO_3 solution and few mg was used for further characterization.

Powder X-ray diffraction (pXRD). pXRD was recorded with a Rigaku MiniFlex 600 with Bragg-Brentano geometry (θ-2θ mode), Cu K_α source (λ = 1.54184 Å, 40 kV/15 mA for X-ray generation), and a D/teX Ultra Si strip detector with a standard detection mode. Measurement was carried out under inert conditions. 1 mg of a precipitate was fixed in between two Kapton tape layers and mounted on the inert gas holder. The acquired data were treated and analyzed on the software PDXL (Version 2.6.1.2, Rigaku) [17] (Figure S9)

Synthesis of PuO_2 NPs and intermediate Pu(V) phase:

Pu(VI) solution was prepared by oxidation of ^{242}Pu (99.74 mass.%) stock solution with $NaBrO_3$ under slight heating. Its valence states were verified by UV-vis spectrometry (TIDAS 100 J&M Analytics). The aliquots of Pu(VI) solution was added to 3 M $NH_3{\cdot}H_2O$ in the volume ratio 1:10 under continuous stirring. Total concentration of Pu in solution was around $6{\cdot}10^{-5}$ M. The pH and Eh value in the system was controlled. After 2 hours of the stirring the solution was centrifuged to collect the intermediate Pu(V) phase (23900 g, 30 min, EBA 12 (Hettich)). The sample was washed one time with Milli-Q water (18.4 MΩ/cm) to get rid of the ammonia presence and sealed in the container for the X-ray measurements. In order to obtain the "equilibrium phase", the synthesis was repeated under the same conditions, but the equilibrium time was 3 weeks. The sample was washed tree times with Milli-Q water (18.4 MΩ/cm) to get rid of the ammonia presence.

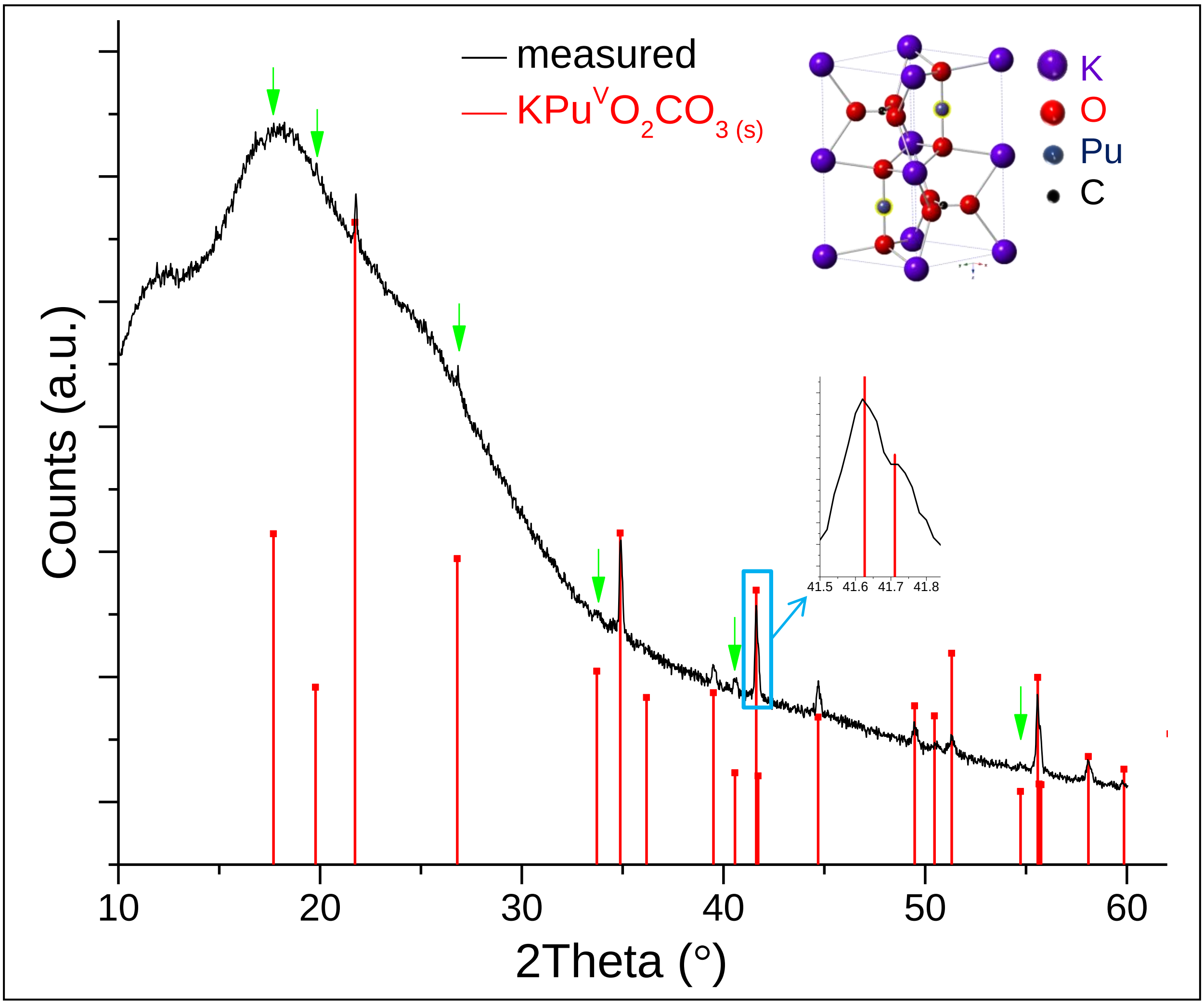

Figure S11. pXRD patterns of Pu precipitated from 0.1M K_2CO_3 solution and reference $KPuO_2CO_{3(s)}$ (ICSD 15685). The unit cell of $KPuO_2CO_3$ is highlighted. High background (up to 30° 2Theta) arises from several Kapton layers used for the sample confinement).

Experiment details:

<u>HERFD at the Pu M_4 edge</u>

The HERFD spectra at the Pu M_4 edge were collected at Beamline ID26 of ESRF[18]. The incident energy was selected using the <111> reflection from a double Si crystal monochromator. Rejection of higher harmonics was achieved by three Si mirrors at an angles of 3.0, 3.5 and 4.0 mrad relative to the incident beam. The Pu HERFD spectra at the M_4 edge were obtained by recording the maximum intensity of the Pu Mβ emission line (~3534 eV) as a function of the incident energy. The emission energy was selected using the <220> reflection of five spherically bent Si crystal analyzers (with 1m bending radius) aligned at 66° Bragg angle. The paths of the incident and emitted X-rays through air were minimized in order to avoid losses in intensity due to absorption. A combined (incident convoluted with emitted) energy resolution of 0.4 eV was obtained

Calculated amounts of $KPu(V)O_2CO_{3(s)}$, intermediate Pu(V) phase and final phase of PuO_2 NPs samples were packed into a specially designed holders consisting of two confinements with 12 and 25 µm Kapton foils as a 1st and 2nd confinement, correspondingly. Holder were then placed

into a gas tight container for the safe transport and used for the U M_4 edge HERFD-XANES measurements at ambient conditions.

HERFD at the Pu L_3 edge

The L_3 HERFD experiments were performed at the Rossendorf Beamline of ESRF. The energy of the X-ray beam was tuned by a double-crystal monochromator operating in channel-cut mode using a Si(111) crystal pair. Two rhodium-coated Si mirrors before and after the monochromator were used to collimate the beam into the monochromator and to reject higher harmonics. XANES spectra were simultaneously measured in total fluorescence yield (TFY) mode with a photodiode and in HERFD mode using an x-ray emission spectrometer.[19] The sample, crystal analyzer and silicon drift detector (Ketek) were positioned on the vertical Rowland circle of 0.5 m diameter. The Pu L_3 spectra were collected by recording the intensity of the Pu $L_{\alpha 1}$ emission line (14282.0 eV) as a function of the incident energy. The emission energy was selected using the [777] reflection of spherically bent Si crystal analyzer (with 0.5 m bending radius) aligned at 75.7^o Bragg angle. In the experiment in January one crystal analyzer was used, the beam size was 200×200 µm, and in April experiment five crystals were used, and the beam size was 100×100 µm. The intensity was normalised to the incident flux. A combined (incident convoluted with emitted) energy resolution of 2.8 eV was obtained as determined by measuring the full width at half maximum (FWHM) of the elastic peak. Samples were packed in round polyethylene holders.

Theory:

FDMNES: Pu L_3 edge

All simulations of Pu L_3 HERFD XANES were performed with the FDMNES code[20,21] . Initially, we selected several compounds containing Pu to identify the structure of the intermediate phase. All compounds were simulated with FDMNES using the same parameters: spin-orbit and relativistic effects were considered; quadrupole and dipole transitions were allowed; a 7Å cluster radius around the absorber was considered for full multiple scattering (FMS) and the Green's functions method was chosen. The convolution parameters were kept below the value needed to match the experimental data in order to better observe the spectral features, especially in the edge region. Specifically, "Gamma_hole" was set to 1.0 eV and "Gamma_max" to 15 eV. The absorber was set to be excited and no self consistent field (SCF) was applied to calculate the potential. With these settings, especially with the Green's function in place of the finite difference method (FDM), the calculations are fast enough to allow the screening of several compounds. The results of the simulations are compared to the experimental spectrum of the intermediate phase in Fig. S3. Simulated spectra have been shifted in energy to match the position of the post-edge feature B. For some structures, the relative position of the post-edge features do not match at all with the data and they can be excluded (d, e, h). Structures f and g presents two post-edge features similar to B and C. However, there is no trace of feature A and the distance between B and C is too large to be consistent with the data. Of the remaining structures, i.e. a, b and c, structure "a" is in reasonable agreement with the data, but it misses feature A, moreover features B and C are too far apart. Structures b and c give very similar results and in good agreement with the data. However, structure c is more likely formed during the synthesis. We therefore selected $NH_4PuO_2CO_3$ as the most promising compound for the intermediate Pu(V) phase. The EXAFS fitting based on the selected structure confirmed the good agreement between the intermediate

Pu(V) phase and $NH_4PuO_2CO_3$ and found a significantly shorter Pu – O distance for the shorter bonds.

Following the EXAFS results, we performed new simulations of the HERFD XANES. We decreased the shorter Pu – O bonds of $NH_4PuO_2CO_3$ and $KPuO_2CO_3$ (from 1.93 Å to 1.82 Å) according to the EXAFS results and we used the FDM method in place of the Green's functions method. The latter needs the muffin tin approximation for the atomic potential, while the FDM method uses a full potential giving substantial improvements especially for the region of the absorption edge. We also simulated the plutonyl(V) aqueous molecule with two distances for the shorter Pu – O bonds: as given by the structural model (1.74 Å) and according to EXAFS (1.82 Å). These simulations are shown in Figure S4 and the results confirm that the structure that better matches the experimental data is that of $NH_4PuO_2CO_3$. The agreement with experimental data improves dramatically and the simulation is in excellent match with the experimental data on the intermediate Pu(V) phase. The "Gamma_hole" parameter was optimized to better match the broadening of the main absorption peak of the data, a final value of 4 eV was used. With the same settings, we simulated the HERFD XANES of bulk PuO2 and the final results are shown in Fig. 3.

Inspection of the partial DOSs elucidate the origin of the spectral features. Figure S5 shows the result of the optimized simulation for $NH_4PuO_2CO_3$. We used here a broadening of 3.0 eV to highlight the features close to the absorption edge. We notice that: the shoulder at the absorption edge is due to transitions to the Pu unoccupied 5f-DOS, while the rest of the spectrum reflects the Pu d-DOS, which is involved in the bonds with neighboring O, C and N ligands. The p-DOS of the closest O, labelled O_{yl}, is strongly hybridized with the Pu d-DOS and is very intense in correspondence of the main absorption peak and of peak B. Equatorial O, C and N are at longer distances and their p-DOSs are mostly participating to feature A. N p-DOS also contributes to peak C.

<u>Anderson Impurity Model (AIM) simulations</u>

The calculations of the Pu M_4 edge spectra were performed in framework of the Anderson impurity model[22]. The spectra of bulk PuO_2 were calculated in a manner described in[23,24] for the Pu(IV) system taking into account the Pu 5f hybridization with the valence states and the full multiplet structure due to intra-atomic and crystal field interactions. The values of the Slater integrals obtained for Pu(IV) using Hartree-Fock formalism were scaled down to 80% to account for the solid state effect. Wybourne's crystal field parameters were set to B^4_0=-0.93 eV and B^6_0=0.35 eV. The ground (final) state of the spectroscopic process was described by a linear combination of the $4f^4$ and $4f^5\underline{\upsilon}^1$ ($3d^94f^5$ and $3d^94f^6\underline{\upsilon}^1$) configurations where $\underline{\upsilon}$ stands for an electronic hole in the valence level. The values for the model parameters were as following: the energy for the electron transfer from the valence band to the unoccupied Pu 5f level Δ=0.8 eV; the 5f-5f Coulomb interaction U_{ff}=5.7 eV; the 3d core hole potential acting on the 5f electron U_{fc}=6.5 eV and the Pu 5f – valence state hybridization term V=1.1 eV (0.9 eV) in the ground (final) state of the spectroscopic process. In calculations for the Pu(V) system, the values of Slater integrals were reduced to 70%. The ground (final) state of the spectroscopic process was described by a linear combination of the $4f^3$ and $4f^4\underline{\upsilon}^1$ ($3d^94f^4$ and $3d^94f^5\underline{\upsilon}^1$) configurations and the value of hybridization term V was increased to 1.2 eV (0.95 eV).

References:

1. Rossberg, A. *et al.* Identification of Uranyl Surface Complexes on Ferrihydrite: Advanced EXAFS Data Analysis and CD-MUSIC Modeling. *Environ. Sci. Technol.* **43**, 1400–1406 (2009).

2. Rossberg, A., Reich, T. & Bernhard, G. Complexation of uranium(VI) with protocatechuic acid?application of iterative transformation factor analysis to EXAFS spectroscopy. *Anal. Bioanal. Chem.* **376**, 631–638 (2003).

3. Pidchenko, I. *et al.* Uranium Redox Transformations after U(VI) Coprecipitation with Magnetite Nanoparticles. *Environ. Sci. Technol.* **51**, 2217–2225 (2017).

4. Newville, M. EXAFS analysis using FEFF and FEFFIT. *J. Synchrotron Radiat.* **8**, 96–100 (2001).

5. Zabinsky, S. I., Rehr, J. J., Ankudinov, A., Albers, R. C. & Eller, M. J. Multiple-scattering calculations of x-ray-absorption spectra. *Phys. Rev. B* **52**, 2995–3009 (1995).

6. Ikeda-Ohno, A. *et al.* Neptunium Carbonato Complexes in Aqueous Solution: An Electrochemical, Spectroscopic, and Quantum Chemical Study. *Inorg. Chem.* **48**, 11779–11787 (2009).

7. Ikeda, A. *et al.* Comparative Study of Uranyl(VI) and -(V) Carbonato Complexes in an Aqueous Solution. *Inorg. Chem.* **46**, 4212–4219 (2007).

8. Scheinost, A. C., Steudtner, R., Hübner, R., Weiss, S. & Bok, F. Neptunium V Retention by Siderite under Anoxic Conditions: Precipitation of NpO 2 –Like Nanoparticles and of Np IV Pentacarbonate. *Environ. Sci. Technol.* **50**, 10413–10420 (2016).

9. Hennig, C., Ikeda-Ohno, A., Emmerling, F., Kraus, W. & Bernhard, G. Comparative investigation of the solution species [U(CO3)5]6− and the crystal structure of Na6[U(CO3)5]·12H2O. *Dalt. Trans.* **39**, 3744 (2010).

10. Ellinger, F. H. & Zachariasen, W. H. The Crystal Structure of KPU0 2 CO 3 , NH 4 PUO 2 CO 3 and RbAMO 2 CO 3. *J. Phys. Chem.* **58**, 405–408 (1954).

11. Dalodière, E. *et al.* Structural and magnetic susceptibility characterization of Pu(<scp>v</scp>) aqua ion using sonochemistry as a facile synthesis method. *Inorg. Chem. Front.* **5**, 100–111 (2018).

12. Di Giandomenico, M. V. *et al.* Structure of early actinides(V) in acidic solutions. *Radiochim. Acta* **97**, (2009).

13. Conradson, S. D. *et al.* Higher Order Speciation Effects on Plutonium L 3 X-ray Absorption Near Edge Spectra. *Inorg. Chem.* **43**, 116–131 (2004).

14. Puigdomenech, I. MEDUSA Software. (2010).

15. Guillaumont, R. *et al.* *Update on the Chemical Thermodynamics of Uranium, Neptunium, Plutonium, Americium and Technetium*. (2003).

16. Simakin, G. A. *et al.* Study of carbonate compounds of pentavalent actinoids with alkali metal cations. *Radiokhimiya* **16**, 859–863 (1974).

17. No Title. *Rigaku J.* **26**, 23–27 (2010).

18. Gauthier, C., Sol, V. A., Signorato, R., Goulon, J. & Moguiline, E. The ESRF beamline ID26 : X-ray absorption on ultra dilute sample. *J. Synchrotron Radiat.* **6**, 164–166 (1999).

19. Kvashnina, K. O. & Scheinost, A. C. A Johann-type X-ray emission spectrometer at the Rossendorf beamline. *J. Synchrotron Radiat.* **23**, 836–841 (2016).

20. Bunău, O. & Joly, Y. Self-consistent aspects of x-ray absorption calculations. *J. Phys. Condens. Matter* **21**, 345501 (2009).

21. Guda, S. A. *et al.* Optimized Finite Difference Method for the Full-Potential XANES Simulations: Application to Molecular Adsorption Geometries in MOFs and Metal–Ligand Intersystem Crossing Transients. *J. Chem. Theory Comput.* **11**, 4512–4521 (2015).

22. Anderson, P. W. Localized Magnetic States in Metals. *Phys. Rev.* **124**, 41–53 (1961).

23. Butorin, S. M., Modin, A., Vegelius, J. R., Kvashnina, K. O. & Shuh, D. K. Probing Chemical Bonding in Uranium Dioxide by Means of High-Resolution X-ray Absorption Spectroscopy. *J. Phys. Chem. C* **120**, 29397–29404 (2016).

24. Butorin, S. M., Kvashnina, K. O., Smith, A. L., Popa, K. & Martin, P. M. Crystal-Field and Covalency Effects in Uranates: An X-ray Spectroscopic Study. *Chem. - A Eur. J.* **22**, 9693–9698 (2016).
